# Autistic Burnout on Reddit: A Sisyphean Struggle with Daily Tasks

**DOI:** 10.1007/s10803-025-06765-4

**Published:** 2025-02-22

**Authors:** Megan M. Clarey, Susan Abel, Michael J. Ireland, Charlotte Brownlow

**Affiliations:** 1 Graduate Research School, University of Southern Queensland, Toowoomba, QLD 4350 Australia; 2 School of Psychology & Wellbeing, University of Southern Queensland, Toowoomba, QLD 4350 Australia; 3 Centre for Health Research, University of Southern Queensland, Toowoomba, QLD 4350 Australia; 4 Australian College of Applied Professions, Sydney, NSW 2000 Australia

**Keywords:** Autistic burnout, Lived experience, Reddit, Qualitative content analysis, Exhaustion, Social withdrawal

## Abstract

The crippling impacts of autistic burnout are well known to the autistic community, yet research is only in its early stages. While research to date has chiefly relied on structured interviews and Delphi studies, it has focused on defining and measuring burnout. What is missing from the research is an analysis of the broader experiences of autistic burnout, and the very real implications that autistic people face when impacted by it. This study reviewed the narratives of autistic people discussing their experiences of autistic burnout on the social media platform Reddit. Using data scraped from Reddit, quantitative and qualitative analyses were undertaken to elicit meaning from the online discourse. After analysing 249 Reddit threads using quantitative content analysis, the results supported existing research identifying three core components of autistic burnout, those being: chronic exhaustion; increased sensory sensitivities; and social withdrawal. New insights were found with users reporting physiological ailments as a complicating factor in their burnout experience. The research also found evidence supporting suggested treatment options for autistic burnout including reducing/stopping social obligations, reducing sensory inputs as much as possible, and time spent alone to reset and recharge. Most importantly, users identified that being autonomous in their recovery choices was critical to the success of their recovery.

## Introduction

Autistic burnout, a profound and often debilitating experience, was thrust into the forefront of research in 2020 by Raymaker et al. However, the term had been appearing within online communities as early as 2008 (Mantzalas et al., 2022), highlighting a significant gap between lived experience and formal research recognition. Despite increased scholarly attention, research into the concept is still in its early stages, and a universally agreed-upon definition is still lacking. This gap underscores the critical need for further investigation to fully understand the nature of autistic burnout, its multifaceted impact on autistic individuals, and potential pathways of recovery.

Through their thematic analysis of interviews and social media posts, Raymaker et al. (2020) proposed that “Autistic burnout is a syndrome conceptualized as resulting from chronic life stress and a mismatch of expectations and abilities with adequate supports. It is characterized by pervasive, long-term (typically 3 + months) exhaustion, loss of function, and reduced tolerance to stimulus” (p. 140). This conceptualization was further supported by Mantzalas et al. (2022), who employed a similar thematic analysis using data from publicly available internet sources. Using a grounded Delphi approach, Higgins et al. (2021) proposed that autistic burnout is “a highly debilitating condition characterised by exhaustion, withdrawal, executive function problems and generally reduced functioning, with increased manifestation of autistic traits” (p. 2356). Higgins et al. (2021) highlight the role of unaccommodating environmental factors, suggesting that burnout is primarily exacerbated by external conditions, contrasting with Mantzalas et al. (2022)’s emphasis on internal challenges such as chronic stress and inadequate support. While the current study focuses on adults, it is worth noting that burnout has also been observed in autistic children and youth. Phung et al. (2021) identified similar characteristics of burnout, including exhaustion, reduced functioning, and emotional overwhelm.

It is important to acknowledge that autistic burnout, while sharing phenomenological similarities with workplace burnout — such as exhaustion, withdrawal, and reduced functioning— is fundamentally distinct in its aetiology and context. Workplace burnout typically arises from chronic job-related stressors and is often alleviated by addressing those specific factors (Maslach & Leiter, 2016). In contrast, autistic burnout stems from the ongoing challenges of navigating a neurotypical-dominated world, with its inherent sensory, social, and communication demands (Higgins et al., 2021; Raymaker et al., 2020). Autistic burnout is influenced by cumulative factors, including systemic and environmental challenges, underscoring the need for sustained personal coping strategies while also advocating for broader societal adaptations to better support autistic individuals (Mantzalas et al., 2022).

Despite ongoing research efforts, autistic burnout remains a nebulous phenomenon, underscoring the critical need for further investigation to fully understand its complexities. Concurrent with efforts to refine the definition of autistic burnout, researchers are also developing tools for its measurement. For instance, Arnold et al. (2023) compared the Academic Autism Spectrum Partnership in Research and Education (AASPIRE) Autistic Burnout Measure (AABM) against their proposed Autistic Burnout Severity Items (ABSI), identifying three core experiences of autistic burnout: exhaustion; withdrawal; and cognitive overload—all evidently influenced by daily life experiences for autistic people. Mantzalas et al. (2024) further explored measurement of autistic burnout by comparing the AABM with the Copenhagen Burnout Inventory – Personal (CBI-P) scale. Their findings support the preliminary validity of both the AABM and the Emotional Exhaustion sub-scale of the CBI-P as tools to assess autistic burnout. Mantzalas et al. also identified exhaustion, withdrawal, and cognitive impacts as key factors, but their research revealed these to be three of seven factors contributing to the experience. Despite advancements in measurement, the conceptual understanding of autistic burnout remains fragmented.

The role of withdrawal in autistic burnout is multifaceted. Raymaker et al. (2020) highlight its function as a recovery method, while Higgins et al. (2021) discuss its manifestation as a symptom. These perspectives underscore the dual nature of withdrawal, illustrating its complexity and the need for further research to explore how these roles intersect.

Previous research on autistic burnout has predominantly employed structured methodologies such as interviews, Delphi studies, and thematic analysis of social media data, which aim to explore and interpret underlying themes. In contrast, our study builds on this foundation by employing naturalistic observation of online discourse through content analysis, a systematic approach that quantifies and categorises content to identify patterns at scale. This approach provides a distinct and complementary contribution to the literature by offering a replicable, structured view of how autistic burnout is conceived, socially constructed (how shared meanings are derived and negotiated), and discussed in spontaneous, free-flowing online interactions. Reddit as a data source provides a unique perspective on the interactions observed among posters and commenters. The capacity for Reddit users to express solidarity and shared experience through comments on posts, while maintaining anonymity, has created an opportunity to quantify the reported experiences of their autistic burnout. This method allows us to capture unfiltered and spontaneous discussions among autistic individuals, providing an authentic representation of their lived experiences. By focusing on these organic conversations, we aim to contribute new insights that may help clarify the fragmented understanding of autistic burnout. This approach has the potential to uncover critical aspects of autistic burnout that structured research designs might overlook, offering a deeper, more ecologically valid perspective on the phenomenon. Ultimately, our findings could enrich the existing body of knowledge, guiding future research, clinical practice, and support strategies for autistic individuals.

## Methods

### Research Design

We conducted a content analysis of posts from the social media platform Reddit, a platform known to attract a disproportionately large number of autistic individuals due to its relative anonymity and community-driven structure (Caldwell-Harris et al., 2023). This platform has previously proven valuable for exploring sensitive health topics (Bunting et al., 2021; Du et al., 2020; Nobles et al., 2018; Pleasure et al., 2022; Sowles et al., 2018) and autism research (Thom-Jones et al., 2024). Research has demonstrated that Reddit serves as a vital platform for autistic adults, who actively utilise it to seek and share information, forge meaningful social connections, and access essential support. The platform’s community-driven structure and relative anonymity make it an indispensable resource during challenging times, offering a unique and valuable space for candid discussions and collective coping strategies (Sowles et al., 2018; Bunting et al., 2021). This makes Reddit an ideal setting for our study, as it enables us to tap into the authentic, lived experiences of autistic individuals as shared in a supportive and engaged community environment.

While many Reddit users assume anonymity, research using Reddit data raises important ethical considerations (Nicmanis et al., 2024; Proferes et al., 2021). Ethics approval was obtained from the host institution’s Human Research Ethics Committee, contingent on the conditions that only publicly available posts were scraped, and that the anonymity of Reddit users was rigorously maintained. Users may not anticipate that their posts could be analysed for research purposes, which raises questions about implicit versus explicit consent. To address this, our data collection was limited to subreddits without specific prohibitions against research and posts that were openly accessible to the public. To protect user anonymity, unique Reddit usernames were not collected during data and a reverse text search was conducted to ensure that direct quotes used in this study could not be traced back to the original Reddit threads.

The potential benefits of this research include amplifying the voices of autistic individuals and contributing to better-informed support strategies for autistic burnout (Nicmanis et al., 2024; Proferes et al., 2021). However, we also recognise potential risks, such as unintended harm through the misrepresentation or misuse of user-generated content. To mitigate these risks, we adhered to rigorous ethical practices, including deidentification of data and transparency in reporting, to ensure the integrity and respectful treatment of user contributions.

### Data Collection

Using Reddit’s keyword search facility, we scraped all publicly available data containing the keywords “autistic burnout”, collecting posts and associated comments. The posts extracted were dated from June 7, 2020, to May 17, 2023. The data scrape did not use a date limit; therefore, the data extracted represented the totality of data available on Reddit at the time of the data scrape. This was achieved by using Reddit’s Application Programming Interface (API) and modifying the standard scripting using Python coding so that the data were extracted in a format where comments associated with a post were kept as discrete units. The unit of analysis was a “thread”, that is the original post and associated comments. The decision to analyse a post with its associated comments was made to capture the full content of user interactions and the depth of their shared experiences with autistic burnout. The data was extracted on May 18, 2023, in CSV format and then saved as an Excel file. Each thread was given a parent numerical identifier for the post and a child numerical identifier for the comment, with the two numbers separated by a period to identify the post and its corresponding comment (e.g., post 37, comment 9 is represented as 37.9).

### Sample Size and Composition

The data scrape elicited 249 parent posts and their associated comments, producing 3975 individual entries for analysis. Data cells that were posted by the Reddit bot or comprising the term “deleted” or “removed” were excluded from the analysis. This resulted in 436 entries being removed yielding 3539 entries for final analysis.

### Coding Process

The data were imported into NVivo 14 (Lumivero, 2023) as individual threads, ensuring that each thread remained a single unit of data. Qualitative content analysis was then conducted to develop the codebook (Hsieh & Shannon, 2005). Guided by the research question, two of the investigators coded 10% of the data. Investigator one was familiar with autistic burnout and a novice coder, whereas investigator two was less familiar with autistic burnout, but experienced with content analysis. The coding process was iterative, and the initial coding results were reviewed collaboratively by the research team. While the two investigators broadly applied the coding protocol in a consistent manner, some differences in interpretation emerged. These differences were discussed and resolved through team discussions, refining the code definitions to ensure clarity and applicability across the dataset. Once agreement was reached, the 44 codes were classified into five overarching categories, and the codebook was ratified.

To verify consistency in applying the refined codebook, the two investigators independently coded an additional 10% of the data. The comparison of these coded datasets showed strong alignment, with any remaining discrepancies addressed collaboratively to further refine the protocol. This process ensured that the finalised codebook could be applied systematically and consistently across the entire dataset. Investigator one then proceeded to code the entire dataset using the finalised codebook.

### Data Analysis

Once the data were coded, both quantitative and qualitative analyses were undertaken. Frequencies and percentages demonstrated the prevalence of individual codes, while quotes from the data illustrate the range of experiences within each code. This approach provides enhanced understanding of the data and a more comprehensive picture of the lived experience of autistic burnout.

## Results

**Fig. 1:**
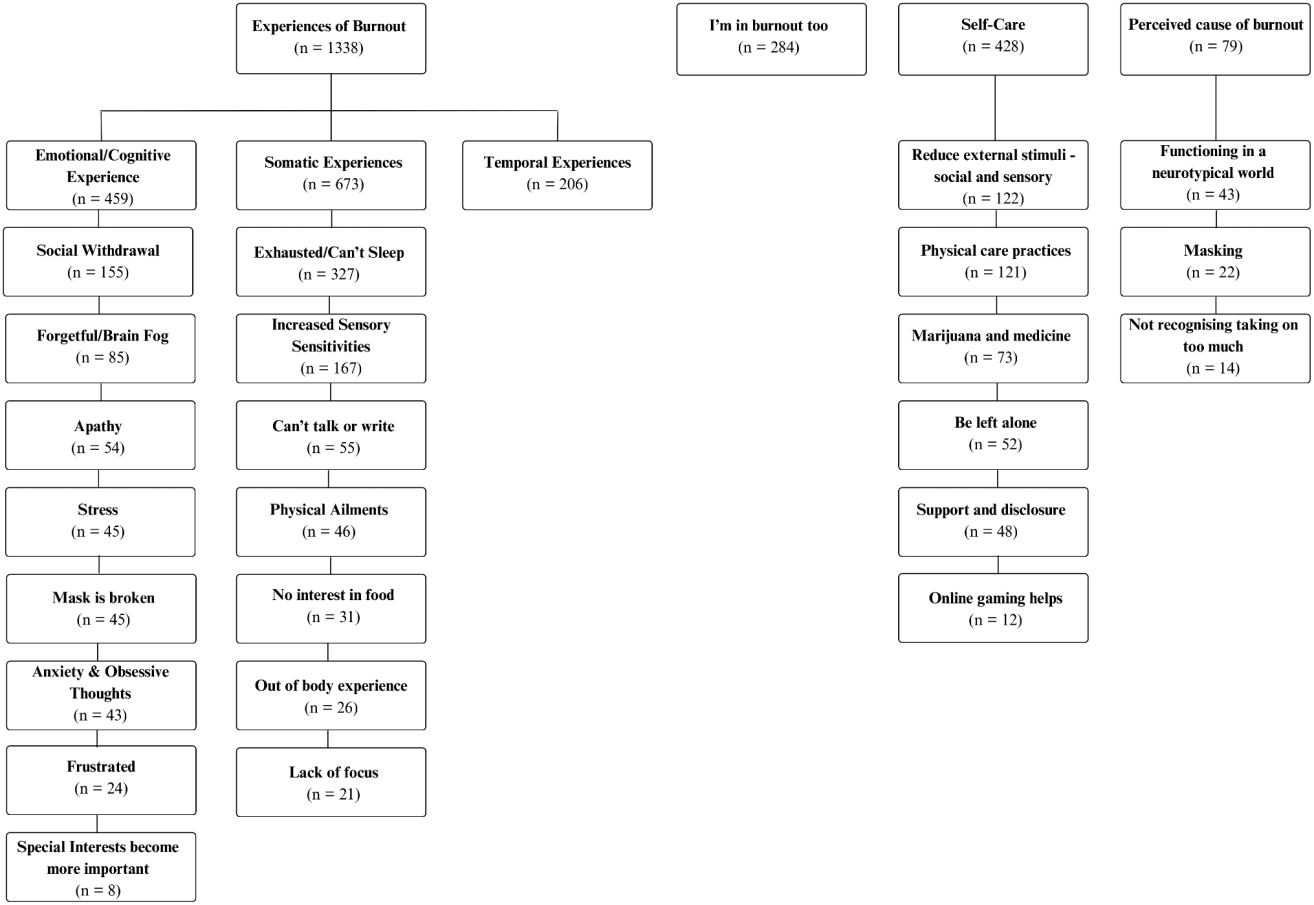
Topic Category, Sub-categories and Codes and associated frequencies developed from Reddit data

The final codebook elicited 44 distinct codes which led to the development of five broad categories: ‘Experiences of burnout’; ‘Response to disclosure’; ‘Self-care’; ‘Perceived cause of burnout’; and ‘I’m in burnout too’ (a phrase frequently used by participants to describe their identification with burnout). The Experiences of burnout category had three sub-categories: ‘Emotional or cognitive experience’; ‘Somatic experience’; and ‘Temporal experience’. The codes and their frequencies are displayed in Fig. 1. Further selections of phrases chosen for codes are found in Table 1 at Appendix A.

### Thematic Insights and Patterns of Autistic Burnout

#### ‘I’m in Burnout Too’ and ‘Temporal Experiences’

User reports of their burnout experiences provided valuable insights, particularly around the themes of shared identification and the duration of burnout episodes. Many users reported both current and historical burnout episodes of varying lengths and intensity. Analysis of these threads revealed a pattern of strong online support, with commenters consistently offering empathy and validation to those sharing their burnout experiences. This pattern was coded as ‘I’m in burnout too’ and was applied when a commentor expressed identification with the state or situation of another user in a thread. In many cases, the original posters and commenters noted how validating it was to discover they were not alone. This theme was coded 284 times, constituting 13.0% of the coded information. Many users regardless of their current burnout status, shared the duration of their burnout episodes. These reported time periods, whether historic or current, were coded as ‘Temporal experiences’ and appeared 206 times representing 9.5% of the coded data. Temporal experiences varied widely, ranging from “it comes and goes in episodes lasting two weeks at a time” (157.4) to “I’ve had this for 25 years and counting” (157.113). Users pinpointed patterns in their burnout episodes such as “I get this a lot but its [sic] more like every 3 weeks I need 1 week to myself to do nothing, and that 3 weeks can be shortened if more days contain activity” (194.209).

Unlike conventional burnout, which is often temporary and linked to specific stressors (e.g., work overload), autistic burnout can extend over decades, persisting in ways that challenge conventional understandings of burnout. Previous research has explored the episodic nature of autistic burnout. Higgins et al. (2021) reported that extended/chronic episodes can occur and noted that shorter periods of up to two weeks may be a warning sign of impending long-term consequences. Mantzalas et al. (2022) also documented autistic burnout periods lasting months or years, highlighting that cumulative experiences increasingly difficult to recover from. The potential for long-term ramifications underscores the need for systems, environments and non-autistic people to understand and provide appropriate support for autistic people recovering from burnout, enabling their successful re-entry into the neurotypical world.

### Experiences of Burnout

#### ‘Exhausted/Can’t Sleep’

The reported experiences of autistic burnout were wide-ranging, encompassing both somatic and emotional/cognitive categories. The most common somatic experience was ‘exhausted or can’t sleep’, coded 327 times. This represents 48.5% of somatic experiences and 28.8% of all coded experiences. Users reported significant sleep and energy dysregulation (“I sleep. I just sleep and sleep and I can’t really do anything else” 157.11), excessive sleepiness (“When I had my burnout…I slept 10 hours during the evening and then 3/4 more during the day” 129.40), disrupted sleep: (“…can’t fight my natural day-sleeping rhythm any more and can’t sleep at night no matter what” 144.5), and persistent exhaustion (“I’ve been exhausted for the last 12 years. Just woke up tired one day and now it’s a personality trait” 194.40).

By combining these experiences under a single code, we aim to reflect the pervasive nature of exhaustion as a multidimensional phenomenon in autistic burnout. While sub-patterns of excessive sleep and inability to sleep can be identified, users frequently described them in conjunction with one another, often emphasising how exhaustion persisted regardless of sleep behaviours. This approach aligns with prior research identifying chronic exhaustion as a core feature of autistic burnout (Raymaker et al. 2020; Higgins et al., 2021) and allows for a holistic understanding of how exhaustion is experienced and expressed.

#### ‘Increased Sensory Sensitivities’

The next most reported experience of burnout was that of sensory sensitivities. Sensitivity to light, noise, temperature, and touch were referenced 167 times comprising 24.8% of somatic experiences and 14.7% of all reported experiences. Notably, hypersensitivity to noise was the most prevalent sensory issue representing over half (85/167) of reported sensory sensitivities. Users described experiences including nausea (“When you talk to me the stimulation makes me nauseous” 144.5), hypersensitivity (“I can hear fluorescent lights just thinking about it” 3.9), and pain (“…noises are making my head hurt but I don’t have a headache” 19.1). Sensitivity to light was also common, representing over a third (60/167) of sensory sensitivities coded. Users described experiences ranging from pain (“every light hurts” 144.5) to hyperawareness (“walking on the stress…everything feels…brighter than usual” 185.2) and avoidance (“my autistic traits such as my aversion to lighting become debilitating” 58.5).

#### ‘Social Withdrawal’

Many users described a need to withdraw from social interactions, both in personal and work settings. This was coded in 33.7% of emotional/cognitive responses and 13.7% of all responses. Slightly more users identified withdrawal from work (81/155) as an aspect of their burnout experience. User experiences related to excessive masking (“My job isn’t physically taxing but I have to do a lot of masking and talking with clients which takes it out of me” 129.6), reduced work capacity (“I had to take two weeks off work, and even now I’m back I’m working at essentially 30% capacity and can barely get that amount done” 157.6), and even resigning (“I had to quit my last job, my dream job because I physically could not hack it. I was grinding myself into the ground”, “I had to quit all my jobs and focus on myself” 194.89 and 2.25). Users reported attempting to implement accommodations in the workplace and having them denied. Examples included: “I asked if I could work 7 hrs a day and they said ‘no we can’t accommodate that, we need you’, so instead of 7 hours a day they get me 0” (154.2), and “…asked for accommodations for 3 12 hour shifts or 4 10 hour shifts…which they said no, so I quit and they got me 0 hours” (154.17).

Social withdrawal manifested in various ways, including withdrawing from groups: “quitting/withdrawing from groups, projects, places, plan” (10.5); limiting interactions: “I do not schedule in person social events any day that I work, and I limit my weekends to no more than two in person social events” (117.2); and even complete withdrawal: “complete cutoff from friends and all social interactions irl [sic] and stop responding to messages” (147.3). For many individuals, the need for withdrawal stemmed from a combination of competing demands, sensory difficulties, and a desire for solitude. This finding, where individuals identify withdrawal as a consequence of burnout, adds nuance to the perspectives of Higgins et al. (2021) and Raymaker et al. (2020), who viewed withdrawal as a potential coping mechanism or a response to social expectations, respectively. While some users described withdrawal as a recovery strategy, others framed it as an involuntary symptom imposed by burnout-related circumstances, such as sensory overload or emotional exhaustion. These perspectives highlight the dual role of withdrawal and its complexity, underscoring the importance of understanding its multifaceted nature in autistic burnout.

Furthermore, users consistently emphasised the involuntary nature of withdrawal and reported significant difficulties in obtaining accommodations to manage their needs. This highlights a potential disconnect between the lived experiences of autistic individuals and prevailing perceptions of withdrawal, emphasizing the need for a deeper understanding of its role in autistic burnout.

#### ‘Forgetful/Brain Fog’, ‘Apathy’ and ‘Can’t Talk or Write’

Another shared experience reported by users was ‘forgetfulness or brain fog’. This experience was described as: “short-thinking (my brain activity seems to only go so far, then stops” (10.5); “kept forgetting what I was saying mid sentence” (157.2); “brain fog, forgetfulness…” (63.5); and “by brain is in a constant fog of confusion” (174.1). This symptom accounted for 18.5% of emotional/cognitive experiences and 7.5% of total experiences. This experience included the loss of skills users had developed over the years. As one user described “OMG, memory loss. The loss of skills. My fingers wouldn’t work. I had difficulty sewing, an activity I’ve done for the last 20 years” (5.18).

Brain fog and forgetfulness are well-documented in the existing research, with Raymaker et al. (2020) and Higgins et al. (2021) both identifying skill loss, confusion, and executive function difficulties as common manifestations. The impact of these cognitive challenges can be extensive, ranging from difficulties with basic daily tasks to the loss of complex, lifelong skills. A particular noted loss was that of being able to talk or write. This was reported 55 times constituting 8.2% of somatic experiences and 4.8% of total experiences. Users reported occurrences of situations such as: “I went from fully functional to not being able to speak or read” (194.190) through to: “I am losing my ability to verbalize” (227.1). In addressing these changes, users also reported a loss of enthusiasm or interest in various activities, further impacting their overall functioning. Coded as ‘Apathy’, this experience represented 11.8% of emotional/cognitive experiences and 4.8% of total experiences. Users reported “No desire to do the things I love” (10.8) and a “Lack of motivation” (157.14). As one user said “I can’t bring myself to draw any more or do other routines. I feel unmotivated or too low energy doing ANYTHING, even though I want to” (13.1).

#### ‘Physical Ailments’

In addition to the somatic experiences already discussed, users also reported various physical ailments that impacted their daily functioning. These included gastrointestinal difficulties, physical pain including joint inflammation and general malaise. This experience represented 6.8% of somatic experiences and 4.1% of total experiences reported. Users posted a variety of instances including “I’m breaking out in hives, my hair is turning grey, my chest hurts, my sciatica is flaring, my stomach is upset” (134.1); “All my skin hurts…also my bones” (144.5); and “I was vomiting 3 to 6 times a day” (47.7). Previous research on autistic burnout has primarily focused on mental well-being and exhaustion, with limited attention to physical health manifestations beyond fatigue (Raymaker et al., 2020; Higgins et al., 2021; Mantzalas et al., 2022). The present findings suggest that physical ailments and illnesses may be exacerbated during burnout periods, highlighting the need to consider their potential contribution to the overall experience of autistic burnout.

#### ‘Stress, Anxiety and Masking’

Within the emotional/cognitive domain, users frequently reported difficulties with ‘Stress’ (9.8%), ‘Anxiety and Obsessive Thoughts’ (9.8%), and ‘Difficulty masking’ (9.8%). Reported occurrences of stress included “Oh boy, I am getting stressed just thinking about it. I get teased about how stressed I get about change in the activities. I dislike how it makes me feel” (19.8); and “everyday, i take at least two one hour (+) breaks to do nothing but what is relaxing” (117.2). While prior research has highlighted a reduced capacity to mask is a common feature of autistic burnout (Raymaker et al., 2020; Higgins et al., 2021; Mantzalas et al., 2022), increased unmanageable stress has not been documented. While increases in anxiety symptoms were noted by Mantzalas et al. (2022), they have not been reported elsewhere. The heightened prevalence of stress and anxiety in our findings suggests that autistic people may experience intensified internalisation of fear and worry during periods of autistic burnout.

#### ‘Frustrated, Lack of Focus and No Interest in Food’

Experiences related to ‘frustration’ (5.2%), ‘lack of focus’ (3.1%) and ‘no interest in food/eating’ (4.6%) were reported. Comments such as “…frustrated with the state of the world” (10.5), and “Oh my god yep my tolerance toward things becomes so low and I find myself really frustrated also it sucks” (142.5) demonstrate the levels of frustration experienced when grappling with autistic burnout. Difficulties with focus were also prevalent, with users stating, “I just can’t maintain any focus or motivation at all” (193.5); and “I now have trouble focusing on anything in my free time” (171.1). Food issues ranged from “I’d practically stopped eating” (194.297) through to “I have ‘can’t eat anything’ periods as well. It goes on for days and it is a downward spiral. The more I go without eating, the weaker I get” (131.16). This grouping emphasises behavioural and functional impacts, consistent with the broader themes of frustration, attentional challenges, and physical well-being disruptions.

#### ‘Out of Body Experiences’

Out of body experiences were also reported by some users, representing 3.9% of somatic experiences. Many users characterised these experiences as “dissociating”. Experiences such as “I experience a total disconnect from my body when my mind is spiraling/panicking [sic]” (72.2); and “Have an out-of-body experience” (10.33) were described. One particularly descriptive comment was “The state that I usually call dissociation is an unpleasant sense of being out of my body and working on autopilot, and this comes on as a result of extreme stress, usually in social situations” (10.34).

The reported out-of-body experiences align with previous research linking dissociation and autistic shutdowns to burnout (Mantzalas et al., 2022). However, as noted in Phung et al. (2021), shutdowns and burnout, while interconnected, are distinct phenomena. Shutdowns are characterised by internal withdrawal and sensory overload responses, often triggered by acute stress. Our findings suggest that the dissociative experiences described by users may reflect elements of both burnout and shutdown, underscoring the nuanced interplay between these phenomena. Additionally, the observed increases in frustration and difficulties with focus are consistent with the documented loss of executive function skills during autistic burnout (Raymaker et al., 2020; Higgins et al., 2021; Mantzalas et al., 2022).

### Self-care

Discussions of self-care and recovery were prevalent among users sharing their burnout experiences, accounting for 19.8% of the coded data within the ‘Self-care’ category. Six codes were identified within this category. Strategies involving the reduction of external stimuli were most frequently mentioned (28.5%), with ‘Reduce external stimulus’ being slightly more prevalent (54.9%) than ‘Reduce social activity’ (45.1%). User comments included: “The best I could come up with was to try to shut out as much stimulus as possible and just get one thing done at a time” (144.13) and “…to get really picky about which social activities I engage in to give myself as much space as possible” (101.2). Some users reported using both methods, demonstrated by comments like “And the burnout starts to feel relieved once I remove sensory problems, stop masking and reduce social interaction for a bit” (63.12).

The next most common self-care methods related to physical care, which represented 28.3% of the self-care codes. Encompassing ‘Physical care – eating and exercising’ (62.0%), ‘Relying on habit to manage’ (24.8%), and ‘Stimming’ (13.2%), these comments highlighted the importance of ‘getting back to basics’ with physical needs and energy regulation through exercise, nutrition, and self-stimulation as strategies for burnout recovery. Some users reported increased awareness of their limitations with food preparation during burnout, and shared shortcuts they employed to address this challenge: “As for food I have things like yoghurt, eggs, cheese, stuff I can either microwave or heat up in the oven. I can’t maintain food prepping” (3.9), highlighting the reduced capacity for food preparation during burnout. Another user stated: “Living off toast, chocolate and baby fruit pouches is fine better eat something than nothing at all” (3.3). While these comments may overlap with reports of reduced interest in eating described in the “Lack of focus” section, their inclusion here reflects a different context. Specifically, these comments emphasise users’ adaptive strategies for addressing burnout-related challenges rather than the disengagement from eating described elsewhere.

The reliance on established routines and habits was also mentioned as a self-care strategy. One user shared, “…i [sic] have a consistent routine I follow at least five days a week, and a ritual every morning with my breakfast” (117.2), emphasizing the value of predictability and structure. Additionally, many comments offered recommendations and encouragement for developing helpful habits, particularly in managing executive dysfunction. For example, one user suggested, “As for executive dysfunction, breaking down your daily studying tasks (or any tasks) into tiny steps could be helpful. Take it one baby step at a time. I like to use visual routine charts, calendars, planners, and habit tracking apps to help me not procrastinate. Also, keep your to do list simple. Since you’re dealing with burnout, it’s best to choose only the most important two or three things to get done and focus only on those. Good luck!” (126.2). Stimming emerged as a valuable self-care tool for managing burnout symptoms. One user poignantly commented, “…find ways to stim that help you regulate and utilize that as a tool rather than it being a bad thing” (46.2).

Users also spoke of using pharmacological methods to assist with the management of their burnout experience. ‘Marijuana’ and ‘Medicine’ comprised 17.2% of self-care with ‘Marijuana’ being reported more often (67.1%) than ‘Medication’ (32.9%). Comments relating to marijuana ranged from “Weed is the only thing that has managed to make me pass as ‘normal’ my whole life” (211.3) to “Ngl [sic], I smoke before I go to work. Makes it so I don’t burn out” (174.5). Medication use comments were mixed with users reporting experiences ranging from “Medication was the only thing that snapped me out of this” (3.96), to “I take Prozac, which is helping put a floor under my bad times so I don’t go quite so far down…” (211.16).

Two more areas noted as helpful in recovering were those of ‘Being left alone’ (12.1%) and ‘Support and disclosure’ (11.2%). While these may appear contradictory, users emphasized the importance of autonomy over their self-care processes for them to be useful. One comment encompassed this well stating “Yes, alone time. Simple and straightforward. Sometimes I need alone time even from my most beloved humans, like one day a week” (144.60). While the need for solitude may be challenging for some to understand, users consistently expressed their need to be left alone, echoing the sentiment, “I just want to be alone, just me, myself and my thoughts. The only things I find myself drawn to are old trees, forests, and running water (waterfalls, streams and rivers), pretty much in that order” (125.3).

In contrast to the need for solitude, users also highlighted the importance of ‘Support and disclosure’. Their experiences varied, with one user sharing, “Talking to loved ones – for me, talking to loves ons [sic] help a lot. Of course, not all the time” (4.2); and “…therapy and supportive family…” (224.4). Users highlighted the value of both ‘Being left alone’ and ‘Support and disclosure’ managing autistic burnout, emphasizing that the effectiveness of these strategies hinged on their ability to exercise autonomy and self-determination in choosing and implementing them. Finally, 2.8% of the coding in self-care related to gaming as a self-care practice coded as ‘Online gaming helps’. This small percentage may reflect its role as a general coping mechanism or leisure activity rather than a targeted burnout intervention. As one user noted “Who DOESN’T play video games to escape? That’s the whole purpose” (12.9).

While recovery and recuperation practices are referred to in the existing literature, there are no empirically supported treatments available for autistic burnout. Our findings align with previously suggest solutions (Raymaker et al., 2020; Higgins et al., 2021), particularly those emphasising load reduction, acceptance and support, health, wellness and self-care and addressing unique autistic needs (Raymaker et al., 2020). This suggests a wealth of knowledge within the autistic community regarding potential recovery pathways, highlighting the importance of incorporating lives experiences into the development of interventions.

### Perceived Cause of Burnout

Some users explicitly reported their perceived causes of burnout, resulting in 79 responses, a mere 3.7% of all coding points. Three categories emerged: ‘Functioning in a neurotypical world’ (54.4%); ‘Masking’ (27.8%); and ‘Not recognising taking on too much’ (17.7%). These three categories align with factors previously identified in the literature as contributing to autistic burnout (Raymaker et al., 2020; Higgins et al., 2021; Mantzalas et al., 2024). Many comments that were coded as ‘Functioning in a neurotypical world’ specifically highlighted the challenges of navigating neurotypical workplaces. As one commentor stated “I just left my 9 − 5 job because I was reaching the point right before I would hit Autistic burnout, which left me hospitalized last time. I’m feeling really down on myself for not being able to keep a full time job like everyone else without being too stressed, and could use some encouragement.” (151.1). One poignant comment reported “But for every measure I deploy, I come back to the main issue. I just don’t know how to handle what’s expected of me to survive under capitalism” (209.1).

Autistic burnout attributed by the user to masking behaviours is best described by this commentor; “My Autism went undiagnosed for 31 years and burnout is what lead me to discover it, so thats [sic] years of not managing autism in a healthy way and heavy masking” (10.12). Users who identified an inability to recognize their limits as a contributing factor to burnout often described this struggle in both personal and professional contexts. One user shared: “One of my biggest regrets is not knowing my limits” (151.3). Although these three areas have been linked to the onset of autistic burnout in existing research (Raymaker et al., 2020; Higgins et al., 2021; Mantzalas et al., 2022), the present findings suggest that users on Reddit tend to focus more on sharing their immediate experiences of burnout and discussing strategies for recuperation and recovery, rather than explicitly addressing its causes. This pattern highlights an important distinction between how burnout is conceptualised in research versus how it is lived and communicated in personal experiences. While clinical and research frameworks necessarily emphasise causal pathways as essential to understanding burnout, our findings suggest that in the midst of burnout experiences, individuals prioritise making sense of their immediate state and seeking paths to recovery. This emphasis on immediate experiences over causal analysis appears to reflect the lived reality of burnout, where understanding “what’s happening to me now” and “how to get better” takes precedence over “what got me here.”

This observation does not imply that the causes of burnout are less important or less worthy of research. Indeed, understanding causal factors remains essential for developing preventative strategies. Rather, the relative lack of discussion about causes on Reddit illuminates how burnout is experienced and processed at a personal level, where immediate challenges and recovery needs naturally dominate attention. This pattern suggests that while causal understanding may be crucial for prevention and clinical intervention, it may be less central to how individuals naturally frame and communicate their direct experiences of burnout. This insight has implications for how we approach both research and support - acknowledging that while causal analysis is vital for prevention, immediate support and recovery strategies may be more aligned with the lived experience of burnout.

## Discussion

This study employed an innovative approach to explore the lived experience of autistic burnout as shared by Reddit users. The current findings not only confirm pre-existing conceptualisations of autistic burnout but also reveal novel insights into previously unreported experiences. Analysing Reddit data provides access to a naturally occurring dataset where users benefit from a degree of anonymity. This relative anonymity allows for more forthright sharing of experiences. Given Reddit is a platform often used by autistic adults to build connections and find solidarity, this openness is reflected in our findings. Notably, autistic Reddit users appear far more interested in sharing their lived experiences and recovery suggestions than in exploring the root cause of burnout.

A common theme for many users was the affirming nature of the interactions with 13.0% of the data coded for users also experiencing burnout. The realisation they are not alone was a prominent theme and the similarity of impacts and issues appeared to be a validating experience for many. A clear demonstration of the chronic and depleting nature of autistic burnout was found in the 9.5% of the responses that related to the duration of burnout periods. Ranging from weeks to decades, users spoke openly of the protracted nature of autistic burnout and the circumstances that impeded their recovery.

The data shows a preponderance of exhaustion and sleep difficulties reported by nearly 30% of users, a core feature echoed in definitions of autistic burnout proposed by Raymaker et al. (2020) and Higgins et al. (2021). Where the results deviate from existing research is specifically found in the experiences of increased sensory sensitivity and the role social withdrawal plays in autistic burnout. Of particular relevance, heightened sensitivity to sensory inputs was experienced nearly 15% of the time, predominantly seen as hypersensitivity to noise and light.

This increase in sensory sensitivities was recorded as the second most common experience for autistic Reddit users. While Raymaker et al. (2020) include reduced tolerance to stimulus as a key characteristic of autistic burnout and Higgins et al. (2021) categorise it as an optional criterion under increased autistic traits,, our findings expand understanding of how sensory challenges manifest during burnout episodes. The Reddit data reveals a broader and more complex picture of sensory experiences beyond reduced tolerance, encompassing heightened sensitivities, increased intensity of sensory overload, and novel manifestations of sensory challenges that may not have been present pre-burnout. Users described sensory experiences as pervasive and multifaceted, with detailed accounts of how various sensory inputs (noise, light, touch) become increasingly overwhelming and disruptive to daily functioning during burnout.

This rich characterisation of sensory experiences in our data builds upon Raymaker et al.‘s foundational work by illuminating the diverse ways sensory challenges intensify and evolve during burnout. While supporting the centrality of sensory aspects in Raymaker’s definition, our findings provide granular insight into how these sensory changes manifest in lived experience, suggesting that burnout may not only reduce tolerance but fundamentally alter sensory processing and responses. This detailed understanding of sensory experiences during burnout has important implications for both theoretical frameworks and practical support strategies. These findings highlight the need to prioritise sensory sensitivities as a core defining feature in both research and support strategies for autistic burnout.

Social withdrawal, covering both work and personal arenas, was the third most prevalent experience of autistic Reddit users in burnout occurring nearly 14% of the time. Many reported having to quit employment to manage their burnout recovery. Notably, participants frequently identified this withdrawal as a key indicator of their burnout experience, often leading to significant consequences such as leaving employment or reducing social engagement to a bare minimum. This stands in stark contrast to the perspectives of Higgins et al. (2021), who framed withdrawal as a potential *recovery* strategy, and Raymaker et al. (2020), who saw it as a *reaction* to social expectations and demands. Our findings, grounded in the lived experiences of autistic individuals, suggest that withdrawal may be a core *symptom* of burnout itself, potentially driven by exhaustion, sensory overload, or a need to conserve depleted cognitive and emotional resources.

Notably, this study identifies the presence of physical ailments and illnesses as a previously unreported aspect of autistic burnout, suggesting potential complicating factors. The only previously reported physical health issues related specifically to symptoms of exhaustion (Mantzalas et al., 2022). Interestingly, despite prior research reporting increased levels of suicidal ideation (Raymaker et al., 2020; Mantzalas et al., 2022; Higgins et al., 2021) such expressions were very rare in our dataset (*n* = 9). This discrepancy warrants further investigation.

### Practical Implications

The insights garnered from this research highlight several key applied, clinical, and practical implications for improving the management of autistic burnout for both autistic individuals and those who support them. Crucially, it is evident that while some general recommendations for support can be made, it is essential that individuals are granted autonomy to determine how they will address their burnout.

Education and advocacy efforts, encompassing both specialized training for professionals and comprehensive community awareness programs, are imperative. Training should be tailored specifically for healthcare providers, support workers and employers to elucidate the unique aspects of autistic burnout and support options. This training should include information on the early signs of autistic burnout and how to distinguish them from other conditions, as well as strategies for implementing appropriate accommodations for compromised sensory profiles. Furthermore, it is vital to include information about effective communicating with autistic people and involve autistic individuals in the development and implementation of relevant supports systems.

Community awareness programs are necessary to foster a deeper understanding of autistic burnout within the broader population. Raising awareness and providing education across professional and public domains through advocacy campaigns, seminars and workshops can contribute to normalising conversations about autist and autistic burnout. Such initiatives can play a crucial role in reducing the stigma associated with autism and autistic burnout.

The development of appropriate workplace accommodations including supportive human resources policies and flexible working arrangements is instrumental in supporting autistic workers. Creating and implementing policies and procedures that cater to the needs of autistic employees is paramount to ensuring their ongoing psychological and physical safety. These policies should encompass access to relevant mental health supports, a streamlined process for accommodation requests related to sensory/stimulation needs, and comprehensive training on neurodiversity and inclusivity. Additionally, providing access to flexible working hours, adaptable workspaces that can be modified for sensory needs, and even work from home options should be strongly encouraged.

Access to such supports would be mutually beneficial for both autistic employees and employers, aiding in the prevention of autistic burnout, fostering more inclusive work practices, and potentially decreasing employment costs over time. The long-term benefits that would likely arise from implementing programs like those suggested would create a more educated, understanding and accepting world for autistic people.

### Limitations

The data that was collected for this research limits the generalisability of the findings. All the posts were available on a social media platform indicating a level of technical competence and accessibility that may not be representative of all autistic adults. Only posts written in English were analysed which also narrows the capacity to generalise these experiences to all autistic adults. This study was not able to identify or collect demographic data and so the demographic composition of Reddit users related to this study is unknown.

### Future Research

The current findings identify a number of opportunities for future research. Deepening our understanding of the core components of autistic burnout such as increased sensory sensitivities is imperative. Research to expand our knowledge of the physical ailments reported such as general ill health, joint and skin inflammations and other physical conditions as a part of the autistic burnout experience would be beneficial. The disparity between prior research and this study in relation to suicidal ideation and suicidality as part of autistic burnout also warrants further investigation. What we don’t know about autistic burnout far outweighs what we do know, and it is essential we continue to analyse this phenomenon for the future health of the autistic community.

**Table 1:** Table of coding information

Topic	Code	Example Quote (brackets indicate post and comment)	Frequency
Experiences of burnout			
Emotional/Cognitive Experience	Anxiety & Obsessive thoughts	I am constantly overwhelmed and anxious (240.1)	43
	Apathy	Just a complete apathy with no ability to start a task, no wish to finish it even if you can get started, ever greater executive dysfunction (47.6)	54
	Forgetful or Brain Fog	I forgot how to read music and play my instruments. I forgot how to speak a language I had studied for 12 years, and was fluent in. I forgot how to crochet. I forgot almost everything I loved. I never recovered those skills. I have never been able to pick up my lifelong hobby of poetry and art. I feel like I lost everything about myself that I loved. (197.2)	85
	Frustrated	Oh my god yep my tolerance towards things becomes so low and I find myself really frustrated also it sucks (142.5)	24
	Mask is broken	Socially I can barely mask. I miss some social cues and can barely hold conversation most days. I’m usually way too exhausted to go anywhere or talk to anyone. So even texts, phone calls and group chats start to stress me out. (63.2)	45
	Social Withdrawal	I don’t care if I don’t have any social interactions with any other living organism.(125.3) I quit my full time job because of burnout. I asked if I could work 7 h a day and they said “no we can’t accommodate that, we need you” So instead of 7 h a day they get me 0 (151.2)	155
	Special interests become more important	I indulged in my special interests (3.6)	8
	Stress	i hit my limit with stressful life situations (129.22)	45
Somatic Experience	Can’t talk or write	I went from fully functional to not being able to speak or read, (194.190)	55
	Exhausted/can’t sleep	Burnout to me feels like dropping all the balls because you’re too tired to keep juggling (10.10) i slept for 23 h last month. didn’t wake up even once in those 23 h. 10 + hours is my burnout norm (10.30)	327
	Physical ailments	physical illness (colds, flu, fungus infections) (10.5) All of my skin hurts… also my bones (144.5)	46
	Lack of focus	suddenly I just can’t focus on things, lack concentration, feel unable to get on with anything (188.3)	21
	No interest in food	no interest whatsoever in food (2.24)	31
	Out of body experience	Feel emotionally numb or detached - Feel little or no pain (10.33)	26
	Increased sensory sensitivities	I can’t step outside because the sun is too bright (203.1) Loud sounds especially are like having icewater dumped on me (147.3) sensitivity to rough fabrics (147.34)	167
Temporal Experience		I call that my normal life… Except it’s 30 + years of exhaustion (194.206) My longest burnout was nearly 10 years. A lot of it was accumulated burnout from before my transition, and then I just hit a wall at one point. (34.9)	206
I’m in burnout too		You are not alone in this (144.13) Wow, this is the best description for what I’ve been going through the past few months, stuff like this is honestly so validating (194.178)	284
Self-Care	Be left alone	Yes, alone time. Simple and straightforward. Sometimes I need alone time even from my most beloved humans. like one day a week XD (144.60)	52
	Physical care practices	I also suggest checking your diet. Try and detox, or eat healthy for a few days. Lots of fresh fruit, veggies, water, and smoothies. Go for walks outside or near beautiful natural areas. Get fresh air and food in your body and some extra rest. (21.7) i have a consistent routine i follow at least five days a week, and a ritual every morning with my breakfast (117.2) Stim away!!!! Legit. Stimming helps (4.9)	121
	Marijuana and medication	I’m currently in the middle of everything horrible. I smoke a lot of weed to try to keep sane and provide a buffer between me and the world. It’s the only reason I’m hanging on as well as I am (4.3) Medication helped me a lot (106.4)	73
	Reduce external stimuli – social and sensory	At home I keep my curtains completely closed, wear comfortable baggy clothes, keep my earplugs in, eat comfort foods, etc. Basically you want to minimize as many sensory input issues as possible. During this time you may be more sensitive, so anything will help. (63.2) But I literally completely hibernated for a couple solid weeks and then played hermit for a month (3.8)	122
	Online gaming helps	Growing up with Asperger’s, video games definitely helped me cope in my younger years. Thanks Ratchet! (12.60)	12
	Support and disclosure	Lean on the people you trust. For me it is my partner. I am still working on not feeling guilty when I need help during a panic attack or doing chores, etc., but it’s getting easier and 1.2 helps me understand that when I’m feeling like a burden, I’m actually not a burden. (4.7)	48
Perceived cause of burnout	Functioning in a neurotypical world	Trying to pass all the time is commonly described as what leads to autistic burnout. It is exhausting to always be the one expected to carry the labor, especially because I commonly find the expectations to be quite ridiculous.(194.800)	43
	Masking	But looking back, needing to mask 99% of the time, with no personal downtime to unwind it took a long term toll (146.2)	22
	Not recognising taking on too much	I decided after that, though, that I really had to watch what I was “willing” to tolerate, and recognized that I’m not really good at recognizing when too much is too much. (144.52)	14
